# Policy paper of the Committee on Ethics and Task Force on Migration and Mental Health: Migration and mental health of migrants, refugees, asylum seekers – Ethical dilemmas and concerns

**DOI:** 10.1192/j.eurpsy.2025.10092

**Published:** 2025-09-22

**Authors:** Meryam Schouler-Ocak, Sofie Bäärnhielm, Ilaria Tarricone, Kristina Adorjan, Jerzy Samochowiec, Egor Chumakov, Apostolos Veizis, Ahmet Tamer Aker, Silvana Galderisi, Sam Tyano, Mariana Pinto da Costa, Thomas Pollmächer, Luís Madeira, Eka Chkonia, Simavi Vahip, Dinesh Bhugra

**Affiliations:** 1 Department of Psychiatry and Neurosciences, Charité Campus Mitte, Charité – Universitätsmedizin Berlin, Germany; 2 Department of Psychiatry and Psychotherapy, Charité at St. Hedwig Hospital, Charité – Universitätsmedizin Berlin, Germany; 3 Department of Clinical Neuroscience, Center for Psychiatry Research, Karolinska Institutet (KI) & Stockholm Health Care Services, Stockholm, Sweden; 4 Transcultural Centre, Region Stockholm, Stockholm, Sweden; 5 Department of Medical and Surgical Sciences, Alma Mater Studiorum—Bologna University, Italy; 6 Institute of Psychiatric Phenomics and Genomics (IPPG), LMU University Hospital, LMU Munich, Munich, Germany; 7 University Hospital of Psychiatry and Psychotherapy, University of Bern, Bern, Switzerland; 8 Department of Psychiatry, Pomeranian Medical University, Szczecin, Poland; 9 Department of Psychiatry and Addiction, St. Petersburg University, St. Petersburg, Russia; 10 INTERSOS Hellas – Humanitarian Organization, Athens, Greece; 11 Trauma and Disaster Mental Health Program, Istanbul Bilgi University, Istanbul, Türkiye; 12 Department of Psychiatry, University of Naples SUN, Naples, Italy; 13 Department of Psychiatry, Tel Aviv University Medical School, Tel Aviv, Israel; 14 King’s College London, London, UK; 15 Institute of Biomedical Sciences Abel Salazar, University of Porto, Porto, Portugal; 16 Center for Mental Health, Klinikum Ingolstadt, Ingolstadt, Germany; 17 Department of Psychiatry, Institute of Preventive Medicine, University of Lisbon, Lisbon, Portugal; 18 Department of Psychiatry, Tbilisi State Medical University, Tbilisi, Georgia; 19 Department of Psychiatry, Ege University, İzmir, Turkey; 20 Mental Health & Cultural Diversity, Centre for Affective Disorders, Kings College, London, UK

**Keywords:** ethical principles and dilemmas, mental health, migrants, recommendations, refugees

## Abstract

**Background:**

International migration is a complex phenomenon of global and historical relevance. It includes voluntary, forced, and workforce migration, shaped by diverse determinants. Push factors comprise war, persecution, and political instability, while pull factors include stability, economic opportunities, education, and favorable living conditions. Forced migration is frequently associated with displacement and a disproportionate burden of mental health disorders, which are urgent yet difficult to address due to structural, cultural, and legal barriers.

**Methods:**

Evidence demonstrates that restricted health care access exacerbates psychiatric disorders, while treatment delays contribute to poorer outcomes. Barriers include administrative limitations, linguistic and cultural differences, stigma, and resource shortages. This policy paper was developed by the Committee on Ethics and the Task Force on Migration and Mental Health of the European Psychiatric Association (EPA). Relevant literature was reviewed and combined with the professional expertise of committee members. The draft was subsequently evaluated by the Publication Committee and the EPA Board, and revised accordingly.

**Results:**

Ethical principles in refugee care are insufficiently implemented in many European countries. Core principles of medical ethics – beneficence, respect for autonomy, non-maleficence, and justice – as well as the obligation to advance psychiatric standards and apply psychiatric expertise for societal benefit, are inconsistently upheld.

**Conclusions:**

The primary duty of physicians is to promote health and well-being through competent, timely, and compassionate care. The EPA therefore advocates coordinated strategies to mitigate the mental health consequences of war, displacement, and trauma, and to secure equitable access to psychiatric services for migrants and refugees.

## Introduction

The principles of medical ethics include beneficence, respect for patient (autonomy), non-maleficence, justice, the imperative to improve psychiatric practice standards, and the application of psychiatric expertise in service to society [1]. This includes striving for equity in the prevention, treatment, and rehabilitation of psychiatric disorders, and ensuring fair treatment for all individuals [2, 3]). In many European countries, healthcare disparities among racialized, minoritized, and ethnic groups, as well as diverse people have been identified as a major public health concern.

International migration is not a single homogenous process, but a universal and historical phenomenon. Migration encompasses various forms, and due to multiple reasons such as forced migration, voluntary migration, workforce migration, economic migration, mobility, and international migration. Pull and push factors play an essential role in migration. Push factors include war, political instability, famine, and drought, while pull factors include political stability, job opportunities, natural resources, better educational, economy, and better climate. A comprehensive review of the literature by Brennan et al. [4] identified various push and pull factors at the macro- (global and national), meso- (professional), and micro- (personal) levels. Interestingly, many factors driving migration to the UK also drive migration from the UK to other countries and are relevant for all high-income countries [5]. These factors include poor working conditions, employment opportunities, better training and development opportunities, better quality of life, desire for a life change, and personal financial gain. Thus, migration can be a voluntary decision influenced by the social, economic, and political contexts of the home country, or enforced by individual persecution, discrimination, war, or famine, or perceived opportunities in the destination country.

Although often used interchangeably by the general public, there are important distinctions between the terms “migrant” and “refugee,” which should be clarified. Both terms have become derogatory in the recent political discourse in many countries, and suggestions have been made that lesser stigmatizing terms, such as one who migrates or one who seeks refuge [6]. While there is no formal legal definition of an international migrant, most experts agree that an international migrant is someone who changes their country of usual residence, irrespective of the reason for migration or legal status (UN). Generally, a distinction is made between short-term or temporary migration, covering movements lasting between 3 and 12 months, and long-term or permanent migration, referring to a change of country of residence for one year or more.

Refugees are individuals who are outside their country of origin due to fear of persecution, conflict, generalized violence, or other circumstances that have seriously disturbed public order and, as a result, require international protection. The refugee definition is found in the 1951 Convention, regional refugee instruments, and the UNHCR’s Statute (United Nations High Commissioner for Refugees).

Migration itself can be voluntary or involuntary and/or forced, i.e., to escape persecution, harassment, or danger, or due to displacement. More people have been forced to flee their homes than ever before, with 110 million individuals displaced worldwide, according to a 2023 report by the United Nations High Commissioner for Refugees [7]. This is the highest number since World War II. Seventy percent of refugees are hosted by neighboring countries, and 40% of all refugees are children. Up to 80% of these refugees live in low- and middle-income countries, which have limited capacity to provide housing, education, and general health and mental health services [7]. Climate change is an increasingly significant factor driving displacement, further exacerbating the vulnerability of those already forced to flee by increasing food and economic insecurity and creating additional barriers to accessing health and social services. [8] Forced displacement from conflict zones has significantly increased in the last decade due to multiple factors [9].

In 2023, 448.8 million people lived in Europe, 27.3 (6%) millions of whom were not European citizens. In addition, 42.4 (9%) million people were born outside Europe [10]. Reasons for living in Europe include family, work, asylum, and education. The employment rate in the EU among the working-age population is higher for EU citizens (77.1%), than for non-EU citizens (61.9%) in 2022. The forcibly displaced and stateless population in Europe is projected to increase by 2% in 2024, reaching 24.9 million people. The war in Ukraine is exacting a brutal toll, with an estimated 5.8 million refugees across the region. With the European Union (EU)‘s Temporary Protection of Ukrainian refugees extended until March 2025, EU States will continue hosting refugees and providing protection and access to vital services, including education, health, and employment [11].

## Mental Health of Migrants and Refugees

Research on the mental health of migrants and refugees has largely focused on the three phases of pre-migration, migration, and post-migration [12], as well as a five-phase model encompassing pre-departure, journey, interception, destination, and return [13]. A majority of forced migrants are internally displaced people who often have significant mental health problems, which, although urgent, are difficult to manage due to numerous obstacles [14]. A growing body of evidence indicates that a large proportion of migrants and refugees suffer from the consequences of traumatic events and develop mental disorders such as post-traumatic stress disorder, depressive and anxiety disorders, and relapses into psychotic episodes [15]. Beyond childhood trauma, there is increasing evidence that bullying, social exclusion, and discrimination during adolescence and adulthood have been linked to an increased risk of developing psychotic disorders. Such forms of trauma may also contribute to the elevated risk of psychosis among migrants or individuals with visible minority status [16]. A substantial increase in the risk of developing non-affective psychosis has been observed in those exposed to trauma or discrimination, with the highest risk associated with vulnerability to ethnic discrimination, as indicated by visible minority status [17, 18].

A recent meta-analysis of international studies on serious mental disorders among refugees and asylum seekers found that the most prevalent disorder was major depressive disorder (MDD) (32%), followed by PTSD (31%), recurrent episodes of MDD (16%), and bipolar disorders (BPD_ (5%). The prevalence of psychotic disorders was 1% [19]. Similar findings were reported in a meta-analysis of research conducted in Germany, with PTSD at 29.9% (95% CI 20.8–38.7%) and depressive symptoms at 39.8% (95% CI 29.8–50.1%) [20]. Another systematic review and meta-analysis by Lindert et al. [21] found that the rate of mental disorders among refugees was twice as high as among economic migrants in Europe, with 44% of refugees suffering from depression, 40% from anxiety disorders, and 36% from PTSD [21]. Similarly, Steel et al. [22] reported prevalence rates of 30.6% for PTSD and 30.8% for depression in adult refugees, compared to rates of 1–12% for both disorders in the general population [23]. These data suggest that nearly one in three people who have experienced conflict suffer from a psychological trauma disorder.

Morina et al. [24] highlighted that civilians affected by war are at an increased risk of mental health problems, including post-traumatic stress disorder (PTSD), anxiety, and depression. Koenen et al. [25], based on the results of a WHO Mental Health Survey, demonstrated that PTSD can be particularly long-lasting in the context of war. The effects of the current crisis on mental health may therefore be enduring [26].

Furthermore, forcibly displaced individuals from countries with severe human rights violations have a higher prevalence of psychopathological symptoms [27]. The consequences can be long-lasting, with studies showing that a history of depression and PTSD increases the risk of dementia [28]. Gender-based violence, which is often underreported due to social stigma and inadequate treatment options, further exacerbates the psychological burden in affected regions, both in the short and long term [29, 30]. This increased risk may not only result from war trauma but may also be influenced by socio-economic factors following displacement [31]. The findings of a cross-sectional study of refugee women who experienced multiple severe traumas related to war in their home countries and danger encountered during their migration suggest that family violence was key to their current mental health problems [32]. Therefore, culturally sensitive assessment and treatment need to place special emphasis on these family dynamics.

In a study conducted in Turkey, Başterzi [33] found that the most common psychological problems among refugees included psychosis, anxiety disorders, PTSD, and depression. Refugee children are also vulnerable to behavioral disorders, addiction, introversion, and tendencies towards crime and violence [34].

Tarricone et al. [35] found that the cumulative effect of social disadvantage before, during, and after migration is associated with an increased likelihood of psychosis in migrants, regardless of ethnicity or length of stay in the host country. Public health initiatives that address the social disadvantages faced by migrants throughout the migration process, as well as post-migration psychological support, could help reduce the excess incidence of psychosis among migrants. A Canadian study also found that a sense of belonging to Canada was a significant predictor of mental health [36].

In a scoping review, Hilario et al. [37] identified that the mental health of young migrants is shaped by a myriad of social and economic factors at individual, family, community, and societal levels. The review highlighted the need for mental health services to focus on first-generation migrant youth and those who migrated before the age of six, to address high levels of stress or the likelihood of mental illness. In addition, the mental health of young people after migration is influenced by their parents’ settlement experiences in terms of employment, household income, and integration. Parental depression, in particular, is consistently associated with poorer mental health outcomes in migrant youth. This suggests that the experiences of young people living with parental mental illness should be a key focus for future research and targeted interventions [37].

## Improving Mental Health Care for Migrants and Refugees

As discussed above, there is considerable research evidence that both migrant and refugee groups are particularly vulnerable to receiving substandard healthcare, partly due to racist and discriminatory attitudes, behaviors, and policies within the health system [16, 38, 39].

Furthermore, having fled war and conflict, endured torture or traumatic events, and living with ongoing uncertainty [40], these individuals often face access barriers to health care and are at high risk of experiencing long-term poor physical and mental health outcomes in their host countries [41]. A meta-analysis and systematic review have highlighted that racism is strongly associated with poor health outcomes, with the relationship particularly strong for mental health, and less so for physical health [42]. Racism can be defined as organized system within societies that cause avoidable and unfair inequalities in power, resources, capacities, and opportunities across racial or ethnic groups [43]. Several studies have highlighted the negative impact of racial discrimination on mental health, particularly concerning the development of affective, psychotic, and substance use disorders [42, 44, 45].

According to Bäärnhielm and Schouler-Ocak [46], there is a significant gap between the mental health care needs of migrants, refugees, and minority groups and the services available to them, emphasizing the need to improve accessibility and adapt systems, services, and interventions. Health professionals play a crucial role in ensuring the quality of care, and their ability to meet new challenges depends on their competence, knowledge, skills, and attitudes toward their patients’ needs. To enhance their capabilities, mental health professionals require training in cultural diversity and structural competence. Cultural competence encompasses professional values, including sensitivity, non-discrimination, and responsiveness to the psychiatric needs of all patients. Psychiatrists may enhance their intercultural performance during the mental health assessment using the Cultural Formulation Interview (CFI) of the DSM-5. It is supportive in clinical practice, where psychiatrists must consider each patient within the context of the patient’s culture and their own cultural values and prejudices [47]. Cultural competence is vital for understanding, treating, and supporting migrant and refugee patients and for addressing racial discrimination. “Cultural competence” is typically described as a multidimensional set of cognitive orientations, cultural knowledge, skills, sensitivities, and attitudes [48]. (Self-)critical reflection, including an examination of internalized prejudices and value hierarchies, is crucial [49]. The development and application of cultural competence appear to be influenced less by organizational characteristics and more by the level of the individual actors. Therefore, in addition to personnel development, appropriate organizational structures and an economic incentive system are required to promote socio-cultural diversity within mental health care systems [49].

## Barriers to Access Mental Health Care

Difficulties in accessing healthcare often exacerbate existing mental disorders [50] and delays in help-seeking can produce poor outcomes. There are a number of factors at play: from language barriers, poor knowledge of the healthcare systems in new settings, stigma of mental illnesses, differing explanatory models, anxieties regarding the perspectives in the host country [51, 52], and so forth Language barriers and intercultural communication challenges are significant obstacles, making it much harder for individuals to access the healthcare system. The availability of qualified interpreters, language and cultural mediators is inconsistent, and they are not regularly utilized and not available. This can lead to misunderstandings, misdiagnoses, and inappropriate treatment, with potentially serious consequences for the individuals involved [51].In intercultural psychotherapy, language plays a crucial role, as many languages lack equivalent terms for various mental disorders [53]. For example, the term “depression” does not exist in many languages, even though sadness and unhappiness are recognized and described [7, 53, 54]. Consequently, psychotherapists and psychiatrists must be sensitive to cultural and contextual communication aspects [55]. Without interpreters, effective communication between professionals and patients from different cultural backgrounds is sometimes impossible. In psychiatry and psychotherapy [47, 53], language competence is a key factor in the utilization of healthcare services by migrants.

Inequalities between migrants and non-migrants in terms of health and access to healthcare services persist in many European countries. Legal barriers make it difficult for refugees and migrants to access healthcare. Economic constraints also play a role, as many migrants cannot afford healthcare costs [56].

In Europe, healthcare delivery and access are, not surprisingly, heterogeneous [56], making comparisons challenging. Data is often lacking, complicating the ability to draw firm conclusions. Countries apply different standards when prioritizing the healthcare needs of refugees, with some focusing on mental health care, preventive care (vaccinations), and long-term care, especially for an aging migrant population [56, 57].

Political, social, and legal stressors can hinder the integration process [58] as well as an understanding of the new cultures and acculturation. Social factors such as grief, culture shock, social exclusion, and a mismatch between the expectations and attitudes of the host country also affect acculturation [53, 59] and insecurities regarding perspectives in the host country can stop people from seeking help [47, 60].

Growing inequalities in resources within and between countries, the construction of border walls and tighter border controls, and a lack of perspectives in the host country further complicate the integration of migrants and refugees [61]. Additionally, public hostility towards migrants and refugees is increasing in many European countries, making them increasingly unwelcome and placing them in a political atmosphere that frightens them. This tightening of reception conditions, driven by fears of terrorism, leads to insecure residence permits, social exclusion, and difficulties in accessing psychosocial care. In this context, ethical principles and the foundations of modern ethical codes are often compromised.

## Asylum Seekers Benefit Act

European Union legislation stipulates that Member States have a legal obligation to provide for the social and health needs of asylum seekers. This includes granting access to healthcare services. In September 2020, the European Commission introduced a New Pact on Migration and Asylum, setting out a fairer approach to managing migration and asylum. The Pact includes provisions for health checks to facilitate the early identification of migrants’ healthcare needs [62]. In Germany, e.g., the social and health benefits available to asylum-seekers are governed by the Asylum Seeker Benefits Act. Asylum-seekers are individuals who have submitted an asylum application and are awaiting the outcome of their asylum determination process [63]. Previously, healthcare entitlement was limited to the first 18 months or until permanent protection status (refugee status or subsidiary protection) was granted. However, this period has now been extended to 36 months. The limited entitlements cover healthcare for acute illnesses and pain, preventive services and immunizations, as well as services related to pregnancy and childbirth (AsylbLG Art. 4). Access to further, mostly specialized, services can be granted on a case-by-case basis (AsylbLG Art. 6).

In contrast, Italy guarantees two constitutional rights to everyone residing in the country, whether permanently or temporarily: healthcare and education. While it may not be possible to address factors before and during migration, post-migration factors can certainly be addressed. Ensuring a dignified welcome for people arriving in the country is therefore a crucial strategy for safeguarding the health of the individual and the wider community.

It is important to note that in many countries and settings, civil society and NGOs often take the lead in demonstrating the social feasibility of innovative social models. These may be influenced by a number of cultural, social, political, and economic factors. Liaising with community organizations and NGOs can help both sides to learn about what is needed and what is available.

Europe is currently hosting millions of forcibly displaced people from Ukraine. Many countries have taken significant steps to enable their rapid integration. With the European Union (EU)‘s Temporary Protection for Ukrainian refugees extended until March 2025, EU Member States will continue hosting refugees and providing protection and access to vital services, including education, healthcare, and employment [11].

The Asylum Seekers Benefits Act has shown that legislation can be amended quickly when necessary. As a result, refugees from Ukraine were granted immediate access to psychiatric care, including special consultation hours with psychiatrists and psychotherapists. Private accommodation was arranged, unemployment benefits were provided from day one in the host countries, work permits were issued promptly, and children were swiftly enrolled in schools. The treatment of Ukrainian refugees illustrates how better conditions are possible and achievable in many countries. Extending these conditions to other asylum seekers could significantly improve their health conditions.

## Ethical Principles in Mental Health Care

The term “ethics” is derived from the ancient Greek word “*ethos*” which refers to the moral norms and values guiding human behavior [64]. This concept encompasses an integrative understanding of morality. The ethical challenges involved in the treatment of migrants and refugees are deeply connected to systemic and cultural issues, as well as to the core principles of professional practice. As Norvoll et al. [65] suggest it is crucial to address the moral aspects of health care and health policy, particularly concerning the treatment of migrants and refugees at individual, structural, and organizational levels.

It is ethically unacceptable to treat people differently on the basis of what the UK calls protected factors such as gender, age, sexual orientation, religion, disability, or ethnic origin. However, access to healthcare for migrants and refugees in many European countries is still very different and regulated by different national laws.

## Specific Ethical Challenges in the Care of Refugees, Asylum Seekers and Migrants

A growing number of immigrants, refugees, and asylum seekers worldwide have limited or no proficiency in the host country’s language(s), which significantly hampers their ability to access regular mental health services. Besides cultural barriers, these language barriers pose significant challenges to medical treatment, especially for those whose access to health care is already limited due to factors such as unfamiliarity with local health care systems or unclear insurance status [47, 66, 67].

Language is the most crucial tool in psychiatry and psychotherapy, making treatment impossible if there is no shared language and no translation between therapist and patient [68]. To facilitate communication, the use of professionally trained interpreters is indispensable.

Moreover, understanding cultural and social norms is essential for accurately interpreting psychiatric and psychological symptoms within the healthcare system. This underscores the importance of diversifying healthcare teams and employing interpreters not only as language mediators but also as cultural mediators. Unfortunately, health insurance companies rarely fund either, which is an untenable situation.

According to the Code of Ethics of the EPA, the fundamental values are

- Responsibility and ethical demands: “Psychiatrists must uphold the responsibilities and ethical demands of the medical profession and specific to psychiatry and working with mental health.
- Non-discrimination: Psychiatrists should consider the ethical principles of respect for autonomy, beneficence, non-maleficence, and justice for all patients regardless of migration status. Psychiatrists shall not discriminate based on age, race, ethnicity, nationality, religion, sex, gender, sexual orientation, social standing, criminal background, disability, disease, political affiliations, or migration status.
- Psychiatrists must never endorse and participate in discriminatory action.
- Cultural sensitivity: Practicing ethical psychiatry requires awareness, sensitivity, and empathy for the patient as an individual, including their cultural values and beliefs.
- Advocacy for Universal Healthcare: Psychiatrists hold the obligation to advocate for universal health care for everyone, and fair and appropriate prevention, care, treatment, and rehabilitation for individuals with mental disorders within available resources in their respective countries.
- Respect and communication: Psychiatrists must stay respectful in communications with patients, patient relatives, and staff. Psychiatrists should promote education of patients, families, and other professionals to empower decision-making processes” [69].
- Decision-making and informed consent: Psychiatrists shall not act as proxy decision makers for their patients and need to stay respectful of patients’ decisions and ensure patients’ rights to express their will (e.g., coercion, restraint, compulsory medication). Psychiatrists should inform patients about diagnostic and therapeutic procedures, promote their autonomy, and always seek their informed consent.
- Rejection of discriminatory practices: Psychiatrist shall not act as a proxy for migration policy.

In communications with patients, psychiatrists must overcome language and cultural barriers, ensuring that they provide security, attention, and adequate time according to the patient’s condition, within available resources. This includes respecting the principles of autonomy and dignity, confidentiality, and appropriate care for gravely ill or disabled patients, including those under involuntary (compulsory) care and treatment.

In many European countries, the ethical principles in dealing with refugees are not taken sufficiently seriously, which contradicts the UN Resolution 46/119 of the Principles for the Protection of Persons with Mental Illness. According to this resolution, psychiatrists should oppose discriminatory practices that limit their benefits and entitlements, deny parity, restrict treatment options, or limit their access to proper medications for patients with mental disorders. In some countries, psychiatry is misused to write reports on the mental health conditions of these migrants, so that they can be deported by the police. This contradicts fundamental values that psychiatrists should act according to and uphold the obligation to advocate for universal health care for everyone, including fair and appropriate prevention, care, treatment, and rehabilitation for migrant and refugee patients with mental disorders within available resources in their respective countries.

The treatment of Ukrainian refugees is a good example of how better conditions are possible and achievable in many countries. Extending these conditions to other asylum seekers could significantly improve their health conditions.

## Recommendations

The primary duty of a physician is to promote the health and well-being of individual patients by providing competent, timely, and compassionate care in accordance with good medical practice and professionalism [70]. Therefore, the EPA recommends strategies to mitigate the impact of war, displacement, and related trauma. According to the aims of this position statement, EPA calls urgently for:

- Action to identify issues related to the mental health of migrants and refugees, bearing in mind that these are heterogenous groups and needs will differ.
- The development of strategies to enable mental health professionals to appropriately treat those severely traumatized by torture, violence, rape, death, and other traumatic experiences.
- Member societies to raise awareness and improve the basic knowledge and skills of psychiatric and mental health professionals regarding the care of migrants and refugees.
- The demand for equal treatment of all migrants and refugees, without any discrimination and exclusion.
- Adherence to the ethical principles when dealing with mentally ill refugees and migrants, in accordance with the UN Resolution 46/119 of the Principles for the Protection of Persons with Mental Illness.
- Commitment to medical ethics, including beneficence, respect for patients (autonomy), non-maleficence, justice, and the imperatives to improve standards of psychiatric practice and to apply psychiatric expertise to the service of society (including seeking equity in prevention, treatment, and rehabilitation of psychiatric disorders) and treating people fairly.

## Data Availability

No publicly available data have been used to support the present paper.
